# S1PR1 regulates NDV-induced IL-1β expression via NLRP3/caspase-1 inflammasome

**DOI:** 10.1186/s13567-022-01078-1

**Published:** 2022-07-19

**Authors:** Pei Gao, Shiyuan Zhang, Xinxin Zhang, Chenggang Xu, Libin Chen, Lei Fan, Jinlian Ren, Qiuyan Lin, Bin Xiang, Tao Ren

**Affiliations:** 1 College of Animal Science and Veterinary Medicine, Henan Institute of Science and Technology, Xinxiang, China; 2 College of Veterinary Medicine, South China Agricultural University, Guangzhou, China; 3 College of Veterinary Medicine, Yunnan Agricultural University, Kunming, China; 4 Postdoctoral Research Base, Henan Institute of Science and Technology, Xinxiang, China; 5 Postdoctoral Research Base, College of Veterinary Medicine, Henan Agricultural University, Zhengzhou, China; 6 Key Laboratory of Animal Vaccine Development, Ministry of Agriculture and Rural Affairs, Guangzhou, China; 7 National and Regional Joint Engineering Laboratory for Medicament of Zoonosis Prevention and Control, Guangzhou, China; 8 Key Laboratory of Zoonosis Prevention and Control of Guangdong Province, Guangzhou, China

**Keywords:** Newcastle disease virus, IL-1β, S1PR1, MAPK, NLRP3 inflammasome

## Abstract

Newcastle disease (ND) is an acute, febrile, and highly contagious disease caused by the Newcastle disease virus (NDV), an important pathogen harmful to domestic poultry. Virulent NDV strain infection induces IL-1β expression and along with strong inflammatory response, ultimately results in death. Inhibition or overexpression of S1PR1, an important target for inflammatory disease treatment, regulates IL-1β expression, suggesting that S1PR1 may alter the degree of the inflammatory response induced by NDV infection by regulating pro-inflammatory cytokine expression. However, the molecular mechanism by which S1PR1 regulates IL-1β expression remains unclear. Here, we explore the expression and tissue distribution of S1PR1 after NDV infection and found that S1PR1 expression increased in the lungs, bursa of Fabricius, and DF-1. IL-1β expression induced by NDV was increased following treatment of cells with the S1PR1-specific agonist, SEW2871. In contrast, IL-1β expression induced by NDV was decreased after cells were treated with the S1PR1 inhibitor W146, suggesting that S1PR1 promotes NDV-induced IL-1β expression. Further investigation demonstrated that NDV induced IL-1β expression through p38, JNK/MAPK, and NLRP3/caspase-1 signaling molecules and S1PR1 affected the expression of IL-1β by activating the NLRP3/caspase-1 inflammasome but had no significant effect on p38 and JNK/MAPK. Our study shows that NDV infection promotes S1PR1 expression and induces IL-1β expression through p38, JNK/MAPK, and NLRP3/caspase-1 inflammasomes and that S1PR1 regulates IL-1β expression mainly through the NLRP3/caspase-1 inflammasome.

## Introduction

Newcastle disease (ND), caused by the Newcastle disease virus (NDV), is an acute, febrile, systemic, and highly contagious infectious disease that endangers poultry [1]. The widespread use of vaccines has mitigated a large-scale outbreak of NDV; however, ND remains a challenge in some areas [2]. After an NDV virulent strain infection, various organs show exudative inflammation and excessive inflammatory cytokine expression, which eventually result in the death of the animal [3–5]. Thus, understanding the signaling pathways that regulate NDV-induced inflammation and identifying appropriate targets to inhibit inflammation not only provide insights into the pathogenic mechanism of NDV but also effective therapeutic approaches against ND.

IL-1β is a central mediator of inflammation that can increase the synthesis and release of IL-8, IL-6, vascular cell adhesion molecules, and intercellular adhesion molecules; cause fever; activate lymphocytes; and promote leukocyte and eosinophil infiltration to the inflammation site [6]. Several signaling pathways can mediate IL-1β production, such as nucleotide-binding oligomerization domain-like receptor protein 3 (NLRP3), mitogen-activated protein kinases (MAPK), and NF-κB. The NLRP3 inflammasome is composed of the NLRP3 scaffold, ASC, and caspase-1, which can regulate the maturation and production of IL-1β [7, 8]. We previously demonstrated that NDV increases IL-1β expression via the NLRP3/caspase-1 inflammasome [9]. MAPK is mainly divided into three pathways: extracellular signal-regulated kinase (ERK), p38, and c-Jun N-terminal kinase (JNK)/MAPK. Various factors can activate the MAPK signaling pathway, including viruses, bacterial toxins, inflammatory factors, and stress, and the activation of MAPK signaling promotes IL-1β expression [10]. NDV activates ERK1/2, p38, and JNK/MAPK signaling molecules and p38/MAPK is associated with NDV-induced cell death [11]. However, the role of MAPK in the NDV-induced inflammatory response remains unknown.

Inhibiting the IL-1β receptor or neutralizing IL-1β significantly reduces the degree of inflammation and pathological damage in the airway caused by the H1N1 subtype influenza A virus (IAV) [12, 13]. We previously determined that neutralizing IL-1β significantly reduces chicken morbidity and mortality caused by the virulent NDV strain, GM [9], suggesting that NDV-induced IL-1β plays a vital role in the pathogenesis of NDV infection and death. Hence, it is necessary to identify suitable targets to inhibit IL-1β production, which will help reduce inflammatory damage caused by NDV.

Sphingosine-1-phosphate receptor 1 (S1PR1) is one of the five G protein-coupled receptors for sphingosine-1-phosphate (S1P) and an important target for treating inflammatory diseases [14]. Walsh et al. used a mouse model to demonstrate that S1PR1 agonists inhibit IAV-induced cytokine storms, reducing the inflammatory response and improving survival rates [15]. Furthermore, S1PR1 activates the NLRP3/caspase-1 inflammasome in mouse macrophages and promotes IL-1β expression [16]. S1PR1 can also regulate inflammatory factor expression by activating the Ras/ERK/MAPK pathway [17, 18]. S1PR1 is involved in the regulation of the MAPK and NLRP3 signaling pathways associated with the production of inflammatory factors caused by viral infection, indicating that S1PR1 may regulate inflammatory responses through the MAPK and NLRP3 signaling pathways. Therefore, exploring the role of S1PR1 in the expression of inflammatory factor IL-1β induced by NDV can provide scientific guidance for the design of NDV prevention strategies.

Our previous study has shown that IL-1β and S1PR1 gene expression levels are significantly increased in SPF chickens and DF-1 cells following infection with GM; S1PR1 overexpression could increase the gene expression of IL-1β induced by NDV. S1PR1 inhibition significantly reduces GM-induced IL-1β gene expression levels [19], suggesting S1PR1 involvement in regulating the GM-induced IL-1β expression process. This study, therefore, explores the role of S1PR1 in the process of NDV-induced IL-1β expression and the main signaling pathway involved.

## Materials and methods

### Cells, animals, and the virus

DF-1 chicken fibroblasts (ATCC CRL-12203) were preserved and cultured in our laboratory. Specific pathogen-free (SPF) chicken embryos (9–12 days) and 8-week-old SPF chickens were obtained from Guangdong Wens Dahuanong Biotechnology Co., Ltd. (Yunfu, China) and housed in micro-isolator cages. The genotype VII virulent NDV strain GM (Chicken/Guangdong/GM/2014) was preserved in our laboratory. Viruses were propagated in 9-day-old SPF embryos, and the allantoic fluid was centrifuged, aliquoted, and stored at −80 °C until use [9]. The median tissue culture infectious dose (TCID_50_) of the virus was determined in DF1 cells using the Reed and Muench method. The viral median egg infectious dose (EID_50_) was measured in SPF chicken embryos.

### Chemicals and antibodies

The S1PR1 specific inhibitor W146 (sc 296700) was purchased from Santa Cruz Biotechnology (CA, USA). The drug-dissolving agent dimethyl sulfoxide (DMSO), S1PR1 specific agonist SEW2871 (S3944), ERK/MAPK-specific inhibitor PD98059 (P215), p38/MAPK specific inhibitor SB202190 (S7067), and JNK/MAPK-specific inhibitor SP600125 (S5567) were purchased from Sigma–Aldrich (St. Louis, MO, USA). Rabbit anti-S1PR1 (EDG-1) antibody (BS2593) was purchased from Bioworld (MN, USA). Rabbit anti-chicken NLRP3 polyclonal antibody was gifted by Zhangyong Ning (South China Agricultural University). Rabbit anti-GAPDH antibody (ab181602) was purchased from Abcam (Cambridge, UK). Rabbit anti-phospho-p44/42 MAPK (Erk1/2) antibody (#9101), rabbit anti-p44/42 MAPK (Erk1/2) antibody (#4695), rabbit anti-phospho-p38/MAPK antibody (#4511), and rabbit anti-p38/MAPK antibody (#8690) were purchased from Cell Signaling Technology (MA, USA). Rabbit anti-phospho-JNK1/MAPK antibody (bs-17591R) was purchased from Bioss Biotechnology Co., Ltd. (Beijing, China). Rabbit anti-JNK1/MAPK antibodies (ab199380) were purchased from Abcam. Rabbit anti-NDV-NP polyclonal antibodies were prepared and stored in our laboratory.

### Animal experiments

Twenty-six 8-week-old SPF chickens were randomly divided into two groups (*n* = 13). The SPF chickens in the GM group were inoculated intranasally (i.n.) with 200 μL 10^5^ EID_50_ of GM virus, and the control group was treated with 0.2 mL of phosphate-buffered saline (PBS). Morbidity and death of the animals were observed daily. On the third day of the experiment, three chickens in each group were slaughtered, and the glandular stomach, lungs, and bursa of Fabricius were collected. Part of the organs was placed in 4% paraformaldehyde for immunohistochemical detection, and the remaining part was frozen at −70 °C for further detection.

### Quantitative real-time polymerase chain reaction (qRT-PCR)

Total RNA was extracted from the glandular stomach, lungs, and bursa of Fabricius using TRIzol reagent (Invitrogen, USA), and ReverTra Ace qPCR RT Master Mix (Toyobo, Japan) was used to reverse transcribe RNA to cDNA. Primers (Table 1) were designed according to the nucleotide sequence of the target gene *NDV M* and *GAPDH* and synthesized by the Sangon Company (Shanghai, China). qRT-PCR was performed in a 7500 Fast Real-Time PCR system using SYBR qPCR mix (Toyobo, Japan) according to the manufacturer’s instructions. The fold change for each gene was calculated using the 2^−ΔΔCT^ method.

**Table 1:** **qRT-PCR primers utilized in this study**.

Primer names	Sequence (5’-3’)	GenBank no
NDV-F	AGTGATGTGCTCGGACCTTC	DQ486859
NDV-R	CCTGAGGAGGCATTTGCTA	
GAPDH-F	CCTCTCTGGCAAAGTCCAAG	V00407
GAPDH-R	CATCTGCCCATTTGATGTTG	

### Immunohistochemical detection

Immunohistochemical detection was performed according to the methods described by Tong [20]. Rabbit anti-S1PR1 polyclonal antibody was used for the immunohistochemical staining of GM-infected tissues from the GM group and uninfected tissues from the control group. The binding of primary antibodies was detected using anti-rabbit HRP (Zhongshan Golden Bridge, Beijing, China). Image J (National Institutes of Health, Bethesda, MD, USA) was used to analyze the mean optical density (MOD) levels of S1PR1.

### Chemical treatment assay and virus infection

The DF-1 cells were seeded into 35-mm cell culture dishes. When the cell confluence reached 90%, the cell culture medium was discarded and washed twice with PBS. Then, DF-1 was pretreated with 1 mL DMSO-dissolved S1PR1 specific agonist SEW2871 (0.5 μM/mL), S1PR1 specific inhibitor W146 (10 μM/mL), ERK/MAPK specific inhibitor PD98059 (10 μM/mL), p38/MAPK specific inhibitor SB202190 (10 μM/mL), or JNK/MAPK specific inhibitor SP600125 (20 μM/mL). Cells with the same volume of DMSO were used as controls. After 1 h of incubation, the original liquid was discarded, and the cells were infected with 1 MOI of GM virus. After 1 h, the virus liquid was discarded, washed twice with PBS, and a cell maintenance solution containing 2% serum was added. Cell culture supernatants and cells were collected 24 h post-infection (hpi) for subsequent experiments.

### Cell viability assay

Cell viability was detected using the CCK-8 cell proliferation and cytotoxicity assay kit (Abmole Bioscience, Houston, TX, USA) according to the manufacturer’s instructions. Cells were grown in 96-well plates at a density of 5 × 10^3^ DF1 cells. After treatment with W146, SEW2871, PD98059, SB202190, SP600125, or DMSO, cells were incubated with 10 μL of CCK-8 for 2 h at 37 °C. The absorbance was measured using a microplate reader at 450 nm with a reference wavelength of 690 nm against the background control.

### Western blot analysis

The cells were lysed on ice with a RIPA buffer containing PMSF and phosphorylase inhibitor, and centrifuged for 10 min at 14 000 × *g* and 4 °C. Then, a BCA Protein Assay Kit (Thermo Fisher Scientific, MA, USA) was used to calculate the protein concentration. The denatured proteins were electrophoresed on a 10% or 12% sodium dodecyl sulfate–polyacrylamide gel electrophoresis gel and transferred to a nitrocellulose (NC) membrane (GE, MA, USA) using the semi-dry blotting method. Then, 10% nonfat milk in TBS containing 0.05% Tween-20 (TBST) was used to block the NC membranes, and the membranes were incubated with antibodies against S1PR1, NDV-NP, p-ERK/MAPK, ERK/MAPK, p-p38/MAPK, p38/MAPK, p-JNK/MAPK, JNK/MAPK, NLRP3, or GAPDH overnight at 4 °C. Next, the membranes were washed in TBST and incubated with IRDye 800CW goat anti-rabbit IgG (1:10 000) for 1 h. After being washed in TBST, the membranes were visualized using the Odyssey Infrared Imaging System (LI-COR Biosciences, Lincoln, NE, USA). Finally, Image J was used to quantify the relative protein levels.

### ELISA

The DF1 supernatant was collected at different time points and centrifuged for 25 min at 5000 × *g* and 4 °C. The concentration of IL-1β was measured using ELISA kits (Cat: SEA563Ga, USCN Sciences Co., Ltd., Wuhan, China) according to the manufacturer’s instructions.

### Caspase-1 activity assay

A caspase-1 activity assay kit (Cat: C1102, Beyotime, Shanghai, China) was used to measure the caspase-1 activity in DF-1 cells according to the manufacturer’s instructions. Briefly, cells were digested with trypsin, and centrifuged for 5 min at 1000 × *g* and 4 °C. After being washed once with PBS, the cell pellet was collected, lysed on ice, and centrifuged for 20 min at 20 000 × *g* and 4 °C. The caspase-1 activity was determined based on the ability of caspase-1 to convert acetyl-Tyr-Val-Ala-Asp p-nitroaniline (AcYVAD-pNA) into p-nitroaniline (pNA) [9].

### Statistical analysis

Data presentation and statistical analyses were performed using GraphPad Prism (version 5.0; GraphPad Software, Inc., La Jolla, CA, USA). Data were expressed as mean ± standard deviation (SD). Data were analyzed using Student *t*-test for pairwise comparisons or an analysis of variance (ANOVA)/Dunn multiple comparison test for multiple comparisons. Statistical significance was set at *p* < 0.05, *p* < 0.01, and *p* < 0.001 for values that were considered significant, very significant, and highly significant, respectively.

## Results

### NDV induces S1PR1 expression in vivo and in vitro

**Figure 1:**
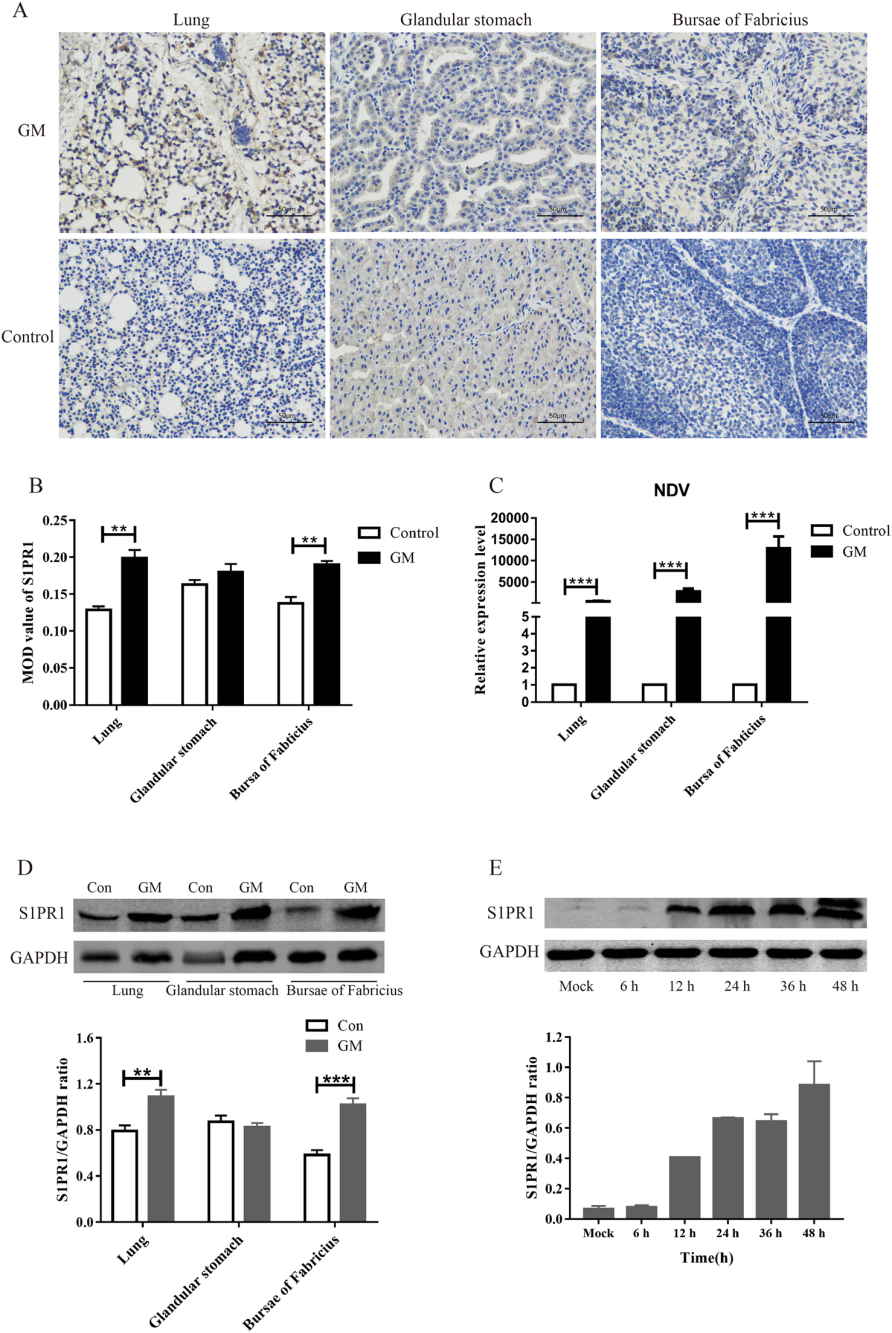
**NDV induced S1PR1 expression in vivo and in vitro.** **A** Immunohistochemical detection of S1PR1 in the chicken’s lungs, glandular stomach, and bursa of Fabricius. GM: GM-infected tissues immunolabeled with the anti-S1PR1 antibody; Control: uninfected tissues immunolabeled with the anti-S1PR1 antibody. **B** The MOD value of S1PR1 in the immunohistochemical image. **C** NDV M gene expression 3 dpi. **D** S1PR1 protein expression in lungs, glandular stomach, and bursa of Fabricius of chickens. **E** S1PR1 protein expression in DF1 cells. Data were expressed as mean ± SD; **P < 0.01 and ***P < 0.001.

The SPF chickens were infected with the genotype VII virulent GM NDV strain or PBS as a control. At 3 days post-infection (dpi), the primary target organs of NDV were collected, including the lung, glandular stomach, and bursa of Fabricius and the tissue distribution of S1PR1 in each organ was detected via immunohistochemistry. S1PR1 distribution in the lung and bursa of Fabricius significantly increased after GM infection (Figures 1A, B). There was no significant difference in S1PR1 expression in the glandular stomach between the GM and control groups (Figures 1A, B). Compared with those in the control, the follicles of the bursa were significantly smaller, the cortex was thinned, the boundary of the cortex and medulla was blurred, and S1PR1 was primarily distributed in the cortex of the bursa following GM infection (Figure 1A). The expression levels of the NDV M gene were determined using qRT-PCR. The gene expression of NDV M was upregulated in the lungs, glandular stomach, and bursa of Fabricius 3 dpi (Figure 1C). Protein samples were obtained and the expression of S1PR1 in each organ was detected using Western blotting. Compared with that in the control group, S1PR1 expression in the lungs and bursa of Fabricius was significantly increased after GM infection (Figure 1D). To test the ability of NDV to induce the expression of S1PR1 protein in vitro, we infected DF-1 cells with GM strain and measured S1PR1 expression 6, 12, 24, 36, and 48 hpi using Western blotting. S1PR1 expression gradually increased with time following infection with GM (Figure 1E), indicating that the virulent NDV strain increases the expression of S1PR1 protein in vivo and in vitro.

### S1PR1 promotes IL-1β expression induced by NDV

**Figure 2:**
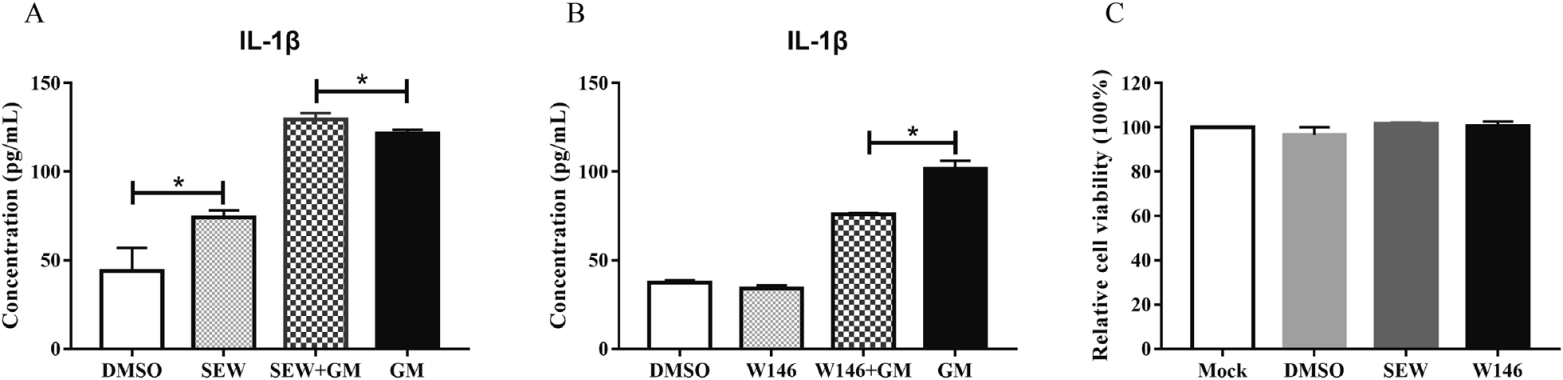
**IL-1β expression and cell viability after S1PR1 agonist SEW2871 or inhibitor W146 treatment.** **A** IL-1β expression after S1PR1 agonist SEW2871 treatment. **B** IL-1β expression after S1PR1 inhibitor W146 treatment. **C** Cell viability after S1PR1 agonist SEW2871 or inhibitor W146 treatment. Similar results were obtained from three independent experiments. Data were expressed as mean ± SD; *P < 0.05.

S1PR1 is a membrane protein receptor that requires activation to function. To verify the regulatory effect of S1PR1 on NDV-induced IL-1β expression, SEW2871, a specific activator of S1PR1, and an S1PR1 inhibitor (W146) were used. Cells were stimulated with SEW2871 and, after 1 h, the original liquid was discarded, and cells were infected with 1 MOI of GM virus. The cell culture supernatant was collected 24 hpi and ELISA was used to measure the expression of IL-1β. The level of IL-1β was 74 pg/mL in the SEW2871 group, which was significantly higher than that in the DMSO control group (Figure 2A). The IL-1β level in the GM-infected group was 121.5 pg/mL, which was significantly lower than that in the group pretreated with SEW2871 (135.5 pg/mL; Figure 2A), indicating that the activation of S1PR1 by SEW2871 increases the expression level of GM-induced IL-1β. The same method was used to detect the effect of W146 on GM-induced IL-1β expression. The IL-1β protein concentration in the GM-infected group was 115.3 pg/mL, which was significantly higher than that in the group pretreated with W146 (75.8 pg/mL; Figure 2B), indicating that S1PR1 promotes the expression of IL-1β induced by NDV. In addition, the treatment of cells with SEW2871 and W146 had no effect on cell viability (Figure 2C).

### The main signaling pathway by which NDV induces IL-1β expression

**Figure 3:**
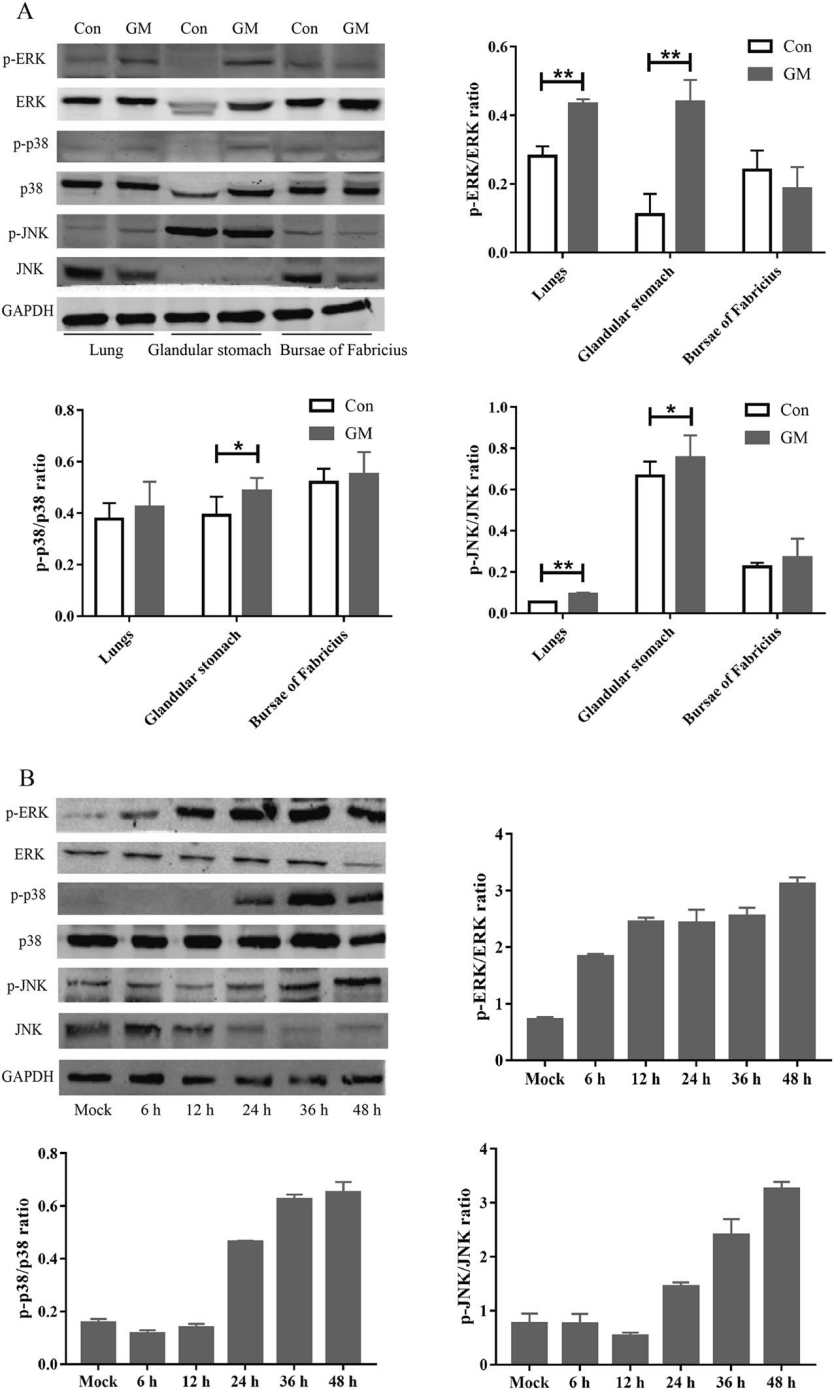
**Activation of ERK, p38, and JNK/MAPK in vitro and in vivo following NDV viral challenge.** **A** Activation of ERK, p38, and JNK/MAPK in the chicken’s lungs, glandular stomach, and bursa of Fabricius. **B** Activation of ERK, p38, and JNK/MAPK in DF1 cells. Similar results were obtained from three independent experiments. Data were expressed as mean ± SD; *P < 0.05 and **P < 0.01.

To explore the signaling pathways by which S1PR1 regulates NDV-induced IL-1β expression, we determined the main signaling pathways involved in the process of NDV-induced IL-1β expression. MAPK and NLRP3 are two important signaling pathways that mediate IL-1β production. We previously demonstrated that NDV induces IL-1β expression through the NLRP3/caspase-1 inflammasome [9]. In this study, the role of the MAPK signaling pathway in the process of NDV-induced IL-1β expression was verified. We first detected the activation of MAPK induced by NDV in vivo and in vitro. The lungs, glandular stomach, and bursa of Fabricius of chickens were collected 3 dpi, and the phosphorylation of ERK, p38, and JNK was detected using Western blotting. After GM infection, the activation levels of ERK, p38, and JNK in the glandular stomach were significantly higher than those in the control group, and the ERK and JNK phosphorylation levels in the lung tissue were significantly higher than those in the control group. In addition, the p38 and JNK phosphorylation levels in the bursa of Fabricius increased, although the difference was not significant (Figure 3A). To determine the activation of MAPK by NDV in vitro, we infected DF-1 cells with GM. The samples were collected 6, 12, 24, 36, and 48 hpi, and the ERK, p38, and JNK/MAPK phosphorylation levels were detected using Western blotting. The unphosphorylated forms of ERK and p38/MAPK were expressed uniformly at each time point, and the level of the unphosphorylated protein of JNK/MAPK gradually decreased with time. The ERK, p38, and JNK phosphorylation levels increased significantly with increasing infection time (Figure 3B), indicating that NDV promotes the activation of ERK, p38, and JNK/MAPK in vitro and in vivo.

**Figure 4:**
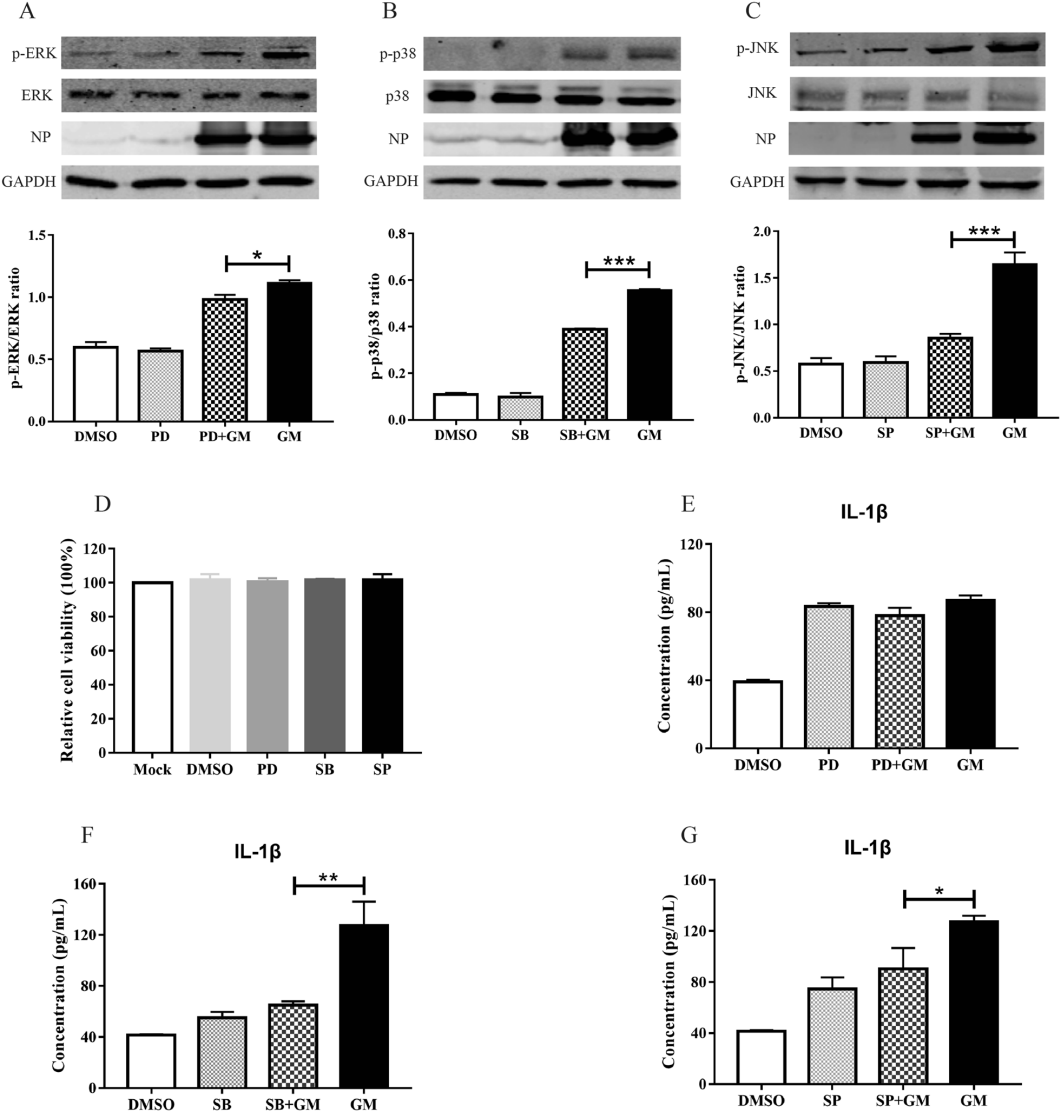
**IL-1β expression and activation of ERK, p38, and JNK/MAPK after ERK, p38, or JNK/MAPK inhibitor treatment**. **A** Activation of ERK/MAPK after PD98059 treatment. **B** Activation of p38/MAPK after SB202190 treatment. **C** Activation of JNK/MAPK after SP600125 treatment. **D** Cell viability after treatment with PD98059, SB202190, or SP600125. **E** IL-1β expression after PD98059 treatment. **F** IL-1β expression after SB202190 treatment. **G** IL-1β expression after SP600125 treatment. Similar results were obtained from three independent experiments. Data were expressed as mean ± SD; *P < 0.05, **P < 0.01, and ***P < 0.001.

To further determine the effect of MAPK signaling molecules on NDV-induced IL-1β expression, we treated cells with PD98059, SB202190, or SP600125 (specific inhibitors of ERK, p38, or JNK/MAPK), infected cells with NDV, and then evaluated their effects on NDV-induced IL-1β expression. Compared with those in the infected group, the ERK, p38, and JNK phosphorylation levels were significantly reduced after treatment with PD98059, SB202190, and SP600125, and the expression of non-phosphorylated proteins of ERK/MAPK was uniform (Figures 4A–C), indicating that PD98059, SB202190, and SP600125 effectively inhibit ERK, p38, and JNK activities. Treatment of cells with PD98059, SB202190, and SP600125 had no effect on cell viability (Figure 4D). IL-1β expression in the cell supernatant was detected using ELISA. The expression of IL-1β induced by GM infection was 78.9 pg/mL 24 h after ERK/MAPK activity was inhibited, and no significant differences were observed compared with the GM-infected control group (86.9 pg/mL; Figure 4E). This indicates that the inhibition of ERK/MAPK signaling molecules does not inhibit GM-induced IL-1β expression. After p38/MAPK activity was inhibited, the expression of IL-1β caused by GM significantly decreased from 113.0 to 64.9 pg/mL (Figure 4F). After JNK/MAPK activity was inhibited with SP600125, the expression level of IL-1β caused by GM infection was 90.5 pg/mL, which was significantly lower than that in the GM-infected group (124.0 pg/mL; Figure 4G), indicating that p38 and JNK/MAPK participate in the expression of IL-1β induced by virulent NDV strains.

### S1PR1 regulates NDV-induced IL-1β expression via NLRP3/caspase-1 signaling molecule

**Figure 5:**
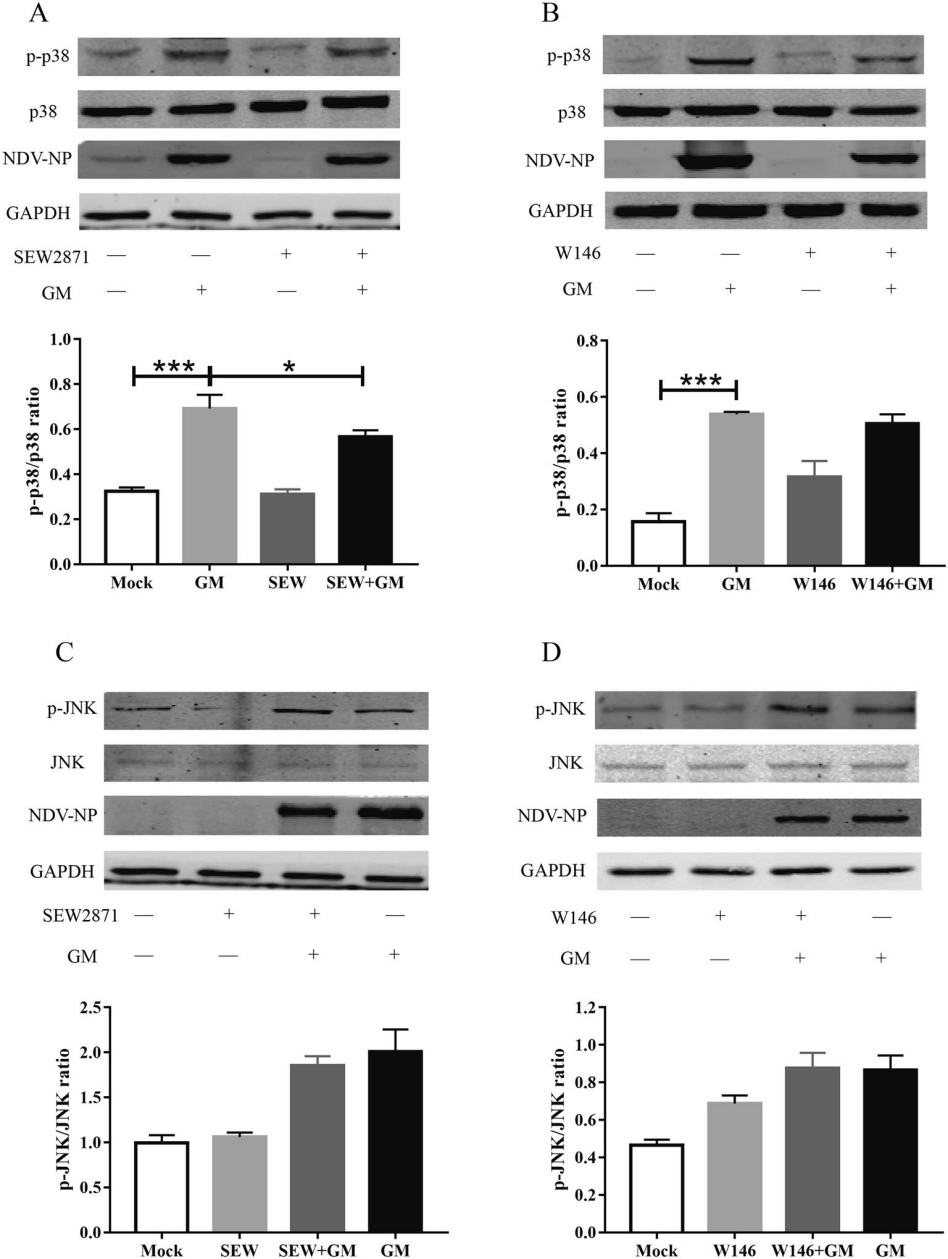
**Activation of p38 and JNK/MAPK following pretreatment with S1PR1 agonist SEW2871 or inhibitor W146.** **A** Activation of p38/MAPK following pretreatment with SEW2871. **B** Activation of p38/MAPK following pretreatment with W146. **C** Activation of JNK/MAPK following pretreatment with SEW2871. **D** Activation of JNK/MAPK following pretreatment with W146. Similar results were obtained from three independent experiments. Data were expressed as mean ± SD; *P < 0.05 and ***P < 0.001.

**Figure 6:**
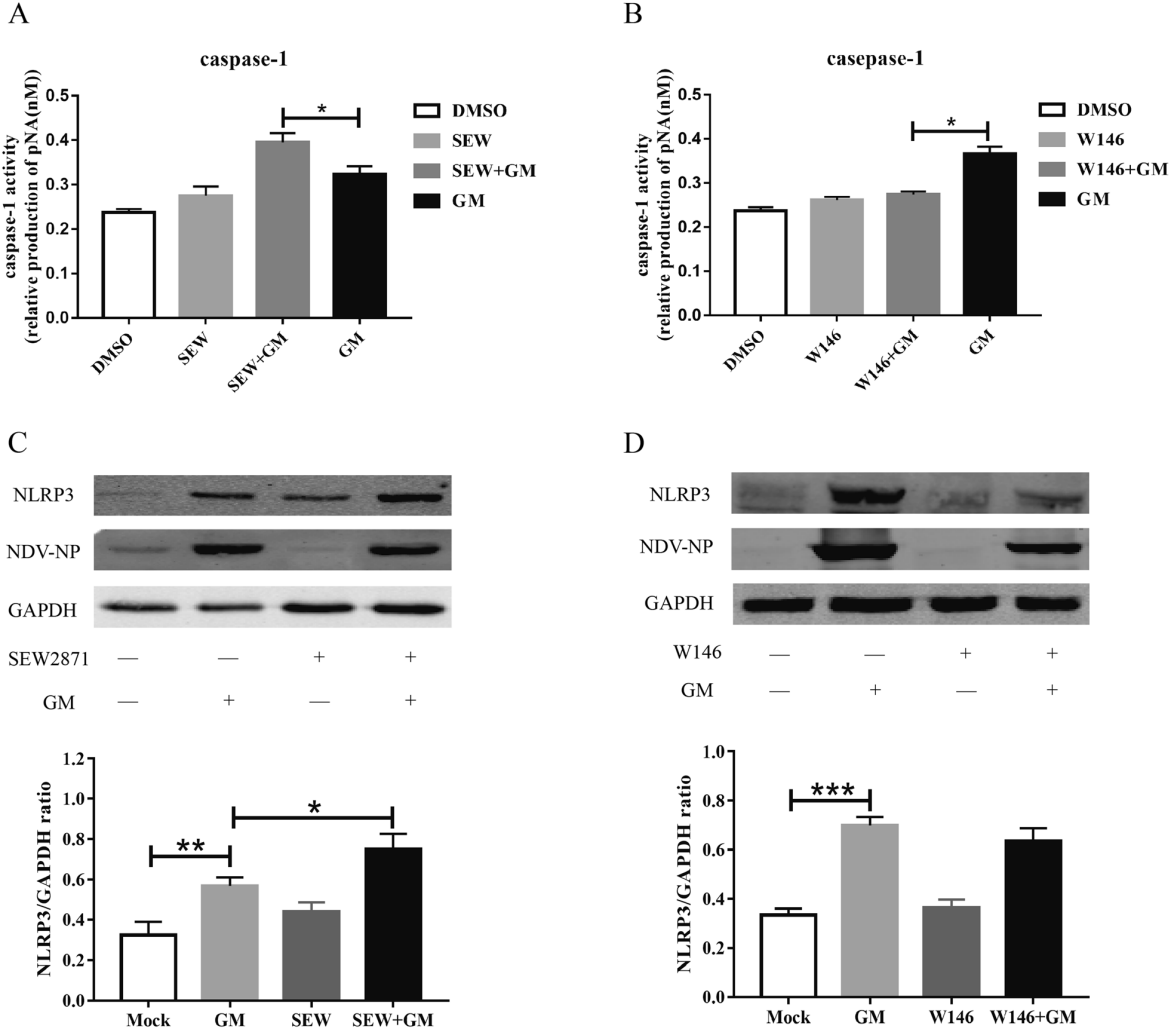
**Caspase-1 activity and NLRP3 expression following pretreatment with S1PR1 agonist SEW2871 or inhibitor W146.** **A** Caspase-1 activity following pretreatment with SEW2871. **B** Caspase-1 activity following pretreatment with W146. **C** NLRP3 expression following pretreatment with SEW2871. **D** NLRP3 expression following pretreatment with W146. Similar results were obtained from three independent experiments. Data were expressed as mean ± SD; *P < 0.05, **P < 0.01, and ***P < 0.001.

The above results suggest that NDV induces the expression of IL-1β through the p38, JNK/MAPK, and NLRP3/caspase-1 signaling pathways. However, further analysis was required to determine the signaling molecules regulated by S1PR1 promoting GM-induced IL-1β expression. Therefore, we examined the effect of S1PR1 on p38, JNK/MAPK, and NLRP3/caspase-1. Cells were stimulated with the S1PR1 agonist SEW2871 or inhibitor W146 and then infected with GM, the phosphorylation levels of p38 and JNK/MAPK were detected using Western blotting. SEW2871 reduced the activation level of p38 and W146 had no significant effect on p38 activation induced by GM (Figures 5A, B). Cells pretreated with either SEW2871 or W146 recorded no significant difference in the activation level of JNK induced by GM (Figures 5C, D). Using the same method, we examined the effect of S1PR1 on the NLRP3/caspase-1 signaling pathway. The activity of caspase-1 induced by GM was 0.39 in the SEW2871 pretreated group, which was significantly higher than that in the GM group (Figure 6A). Following treatment with the inhibitor W146, the GM-induced caspase-1 activity was reduced from 0.37 to 0.28 (Figure 6B). In addition, GM infection induced the expression of NLRP3, and SEW2871 stimulation significantly increased the expression level of NLRP3 induced by GM (Figure 6C). Although not significant (*P* = 0.083), pairwise comparisons showed that the expression of NLRP3 protein in the W146 + GM group was lower than that in the GM group (Figure 6D), indicating that S1PR1 regulates NDV-induced IL-1β expression through the NLRP3/caspase-1 signaling pathway.

## Discussion

The exacerbated inflammatory response induced by NDV infection enhances the pathology in lymphoid tissues and organs, which leads to the death of the host [21]. Identifying potential factors that trigger such harmful reactions and developing therapeutics to inhibit such factors will provide viable therapy for inflammatory damage caused by NDV. We previously determined that neutralizing IL-1β reduces the morbidity and mortality caused by GM [9], indicating that the substantial level of IL-1β expression induced by NDV is an important cause of poultry death. Therefore, an important strategy for controlling NDV is to clarify the mechanism of NDV-induced IL-1β expression and identify suitable targets to inhibit the occurrence of inflammation.

Viral infections can trigger IL-1β expression by activating the NLRP3 inflammasome or MAPK signaling pathway, while different viruses may induce IL-1β expression through different signaling molecules. IAV infection can activate the MAPK signaling pathway and inhibit the MAPK signaling pathway, thereby significantly reducing the expression of inflammatory cytokines caused by AIV [22]. Quercetin inhibits IL-1β-induced inflammatory cytokine expression via the MAPK signaling pathway [23]. In lung fibroblasts, p38/MAPK signaling inhibition significantly suppresses parainfluenza virus-induced IL-1β expression [24]. This study determined that NDV GM strains could activate ERK, p38, and JNK/MAPK in vitro and in vivo, and p38 and JNK/MAPK play important roles in IL-1β expression induced by NDV. Coupled with our previous findings that NDV activates the NLRP3/caspase-1 inflammasome and promotes IL-1β expression [9], we identified that the NLRP3/caspase-1 inflammasome, p38, and JNK/MAPK mediate NDV-induced IL-1β expression. Therefore, we focused on these key signaling molecules and explored targets that can effectively block them.

S1PR1 is an important receptor that regulates inflammation and its role in inhibiting inflammatory responses has been confirmed [16, 25]. Viral infection can induce the expression of S1PR1 in endothelial cells [26]. In this study, the protein expression and tissue distribution of S1PR1 in organs were detected, and the result show that S1PR1 protein expression in the lung and bursa of Fabricius was significantly increased following GM infection compared with that in the control group, which was consistent with our previous results [20], suggesting that S1PR1 is involved in the pathogenic process of NDV.

Our earlier study initially explored the effect of S1PR1 on NDV-induced inflammatory cytokines at the gene level and found that the overexpression of S1PR1 enhances IL-1β expression, while W146 inhibits IL-1β, IL-6, and IL-18 gene expression [20]. To further determine the role of S1PR1 in NDV-induced inflammatory responses, here, the S1PR1-specific agonist SEW2871 was used to treat cells; NDV-induced IL-1β protein expression was significantly increased. After treatment with W146, the protein expression of IL-1β significantly decreased, which confirmed the regulatory effect of S1PR1 on NDV-induced IL-1β expression.

Although S1PR1 is involved in regulating inflammatory cell metastasis and the expression of inflammatory cytokines via different mechanisms [27, 28]. S1PR1 inhibits JNK signaling, promotes apoptosis in myeloid leukemia cells, and promotes cell proliferation by inhibiting ROS production, accelerating elimination, and activating ERK signaling [29]. The activation of S1PR1 with SEW2871 leads to the development of mechano-allodynia by activating the NLRP3 inflammasome; the functional S1PR1 antagonist FTY720 blocks the activation of NLRP3 and production of IL-1β [30]. In rodents, the intrathecal injection of SEW2871 activates the NLRP3 inflammasome, increases IL-1β levels, and causes behavioral hypersensitivity [31]. These previous findings suggest that S1PR1 regulates inflammatory diseases via signaling pathways such as NLRP3 and MAPK; however, the specific regulatory processes vary.

**Figure 7:**
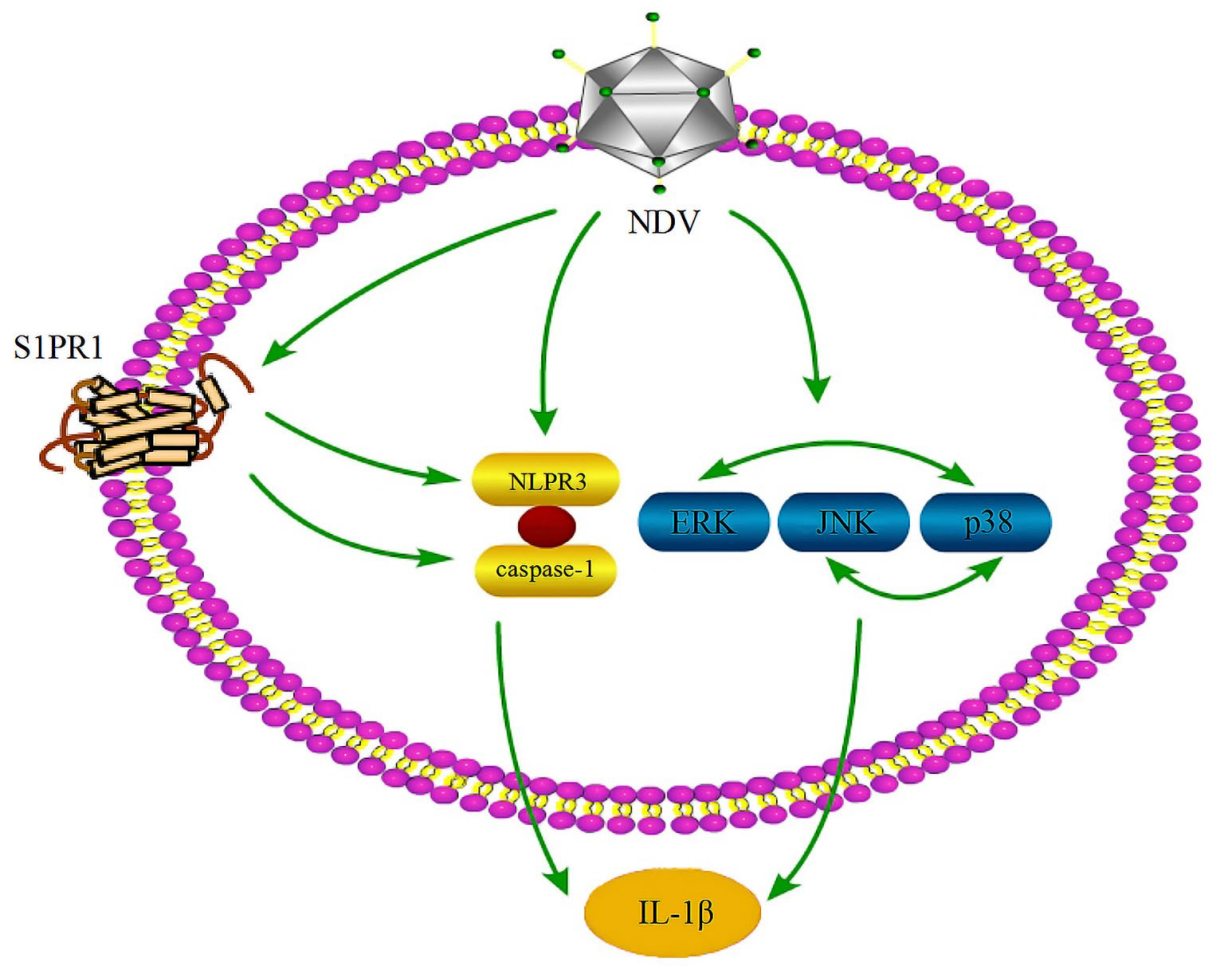
**NDV induces S1PR1 expression and promotes IL-1β expression through the NLRP3/caspase-1 inflammasome**.

To further explain the mechanism by which S1PR1 regulates IL-1β expression, we explored the activation of p38, JNK/MAPK, and the NLRP3/caspase-1 inflammasome after treatment with SEW2871 or W146. S1PR1 had no significant regulatory effect on p38 or JNK/MAPK, whereas after activating S1PR1 with SEW2871, the expression of NLRP3 and activity of caspase-1 significantly increased. After inhibiting S1PR1, NLRP3 expression and caspase-1 activity significantly decreased, indicating that the regulation of S1PR1 on NDV-induced IL-1β expression occurs through the NLRP3/caspase-1 inflammasome. That is, NDV infection activated the NLRP3/caspase-1 inflammasome and MAPK and increased the expression of IL-1β via the NLRP3/caspase-1 inflammasome, p38, and JNK/MAPK. Moreover, NDV promoted the expression of S1PR1, and S1PR1 activated the NLRP3/caspase-1 inflammasome, which increased the expression of IL-1β (Figure 7). In addition to mediating the expression of IL-1β, NLRP3 is associated with the expression of other inflammatory cytokines, such as IL-18 [32], all of which are components of inflammation. S1PR1 can regulate the activation of the NLRP3 inflammasome, suggesting that S1PR1 may affect the degree of the NDV-induced inflammatory response by regulating NLRP3.

The present study reveals a close relationship between S1PR1 and NDV-induced inflammatory disease. However, determining whether S1PR1 has a regulatory effect on inflammatory cytokines, such as IL-6 and IL-18, and verifying how S1PR1 regulates NDV-induced inflammatory responses in chickens warrant further investigations. This will expand our understanding of the therapeutic potential of S1PR1 for NDV.

In conclusion, we demonstrate that NDV induces IL-1β expression via p38, JNK/MAPK, and NLRP3/caspase-1 inflammasomes, and NDV infection promotes S1PR1 expression both in vitro and in vivo. In turn, this enhances NLRP3/caspase-1 inflammasome activity and increases NDV-induced IL-1β expression. These results provide insights into the molecular mechanism by which S1PR1 regulates the inflammatory response, and S1PR1 may be a potential target for the treatment of ND.

## References

[1] Sinkovics JG, Horvath JC (2000). Newcastle disease virus (NDV): brief history of its oncolytic strains. J Clin Virol.

[2] Kang Y, Li Y, Yuan R, Li X, Sun M, Wang Z, Feng M, Jiao P, Ren T (2014). Phylogenetic relationships and pathogenicity variation of two Newcastle disease viruses isolated from domestic ducks in Southern China. Virol J.

[3] Hu Z, Hu J, Hu S, Song Q, Ding P, Zhu J, Liu X, Wang X, Liu X (2015). High levels of virus replication and an intense inflammatory response contribute to the severe pathology in lymphoid tissues caused by Newcastle disease virus genotype VIId. Arch Virol.

[4] Li R, Guo K, Liu C, Wang J, Tan D, Han X, Tang C, Zhang Y, Wang J (2016). Strong inflammatory responses and apoptosis in the oviducts of egg-laying hens caused by genotype VIId Newcastle disease virus. BMC Vet Res.

[5] Rue CA, Susta L, Cornax I, Brown CC, Kapczynski DR, Suarez DL, King DJ, Miller PJ, Afonso CL (2011). Virulent Newcastle disease virus elicits a strong innate immune response in chickens. J Gen Virol.

[6] Peiró C, Lorenzo Ó, Carraro R, Sánchez-Ferrer CF (2017). IL-1β inhibition in cardiovascular complications associated to diabetes mellitus. Front Pharmacol.

[7] Li R, Lin J, Hou X, Han S, Weng H, Xu T, Li N, Chai T, Wei L (2018). Characterization and roles of Cherry Valley duck NLRP3 in innate immunity during avian pathogenic *Escherichia coli* infection. Front Immunol.

[8] Wang B, Zhu J, Li D, Wang Y, Zhan Y, Tan L, Qiu X, Sun Y, Song C, Meng C, Ying L, Xiang M, Meng G, Ding C (2016). Newcastle disease virus infection induces activation of the NLRP3 inflammasome. Virology.

[9] Gao P, Chen L, Fan L, Ren J, Du H, Sun M, Li Y, Xie P, Lin Q, Liao M, Xu C, Ning Z, Ding C, Xiang B, Ren T (2020). Newcastle disease virus RNA-induced IL-1β expression via the NLRP3/caspase-1 inflammasome. Vet Res.

[10] Xing Z, Cardona CJ, Anunciacion J, Adams S, Dao N (2010). Roles of the ERK MAPK in the regulation of proinflammatory and apoptotic responses in chicken macrophages infected with H9N2 avian influenza virus. J Gen Virol.

[11] Bian J, Wang K, Kong X, Liu H, Chen F, Hu M, Zhang X, Jiao X, Ge B, Wu Y, Meng S (2011). Caspase- and p38-MAPK-dependent induction of apoptosis in A549 lung cancer cells by Newcastle disease virus. Arch Virol.

[12] Bucher H, Mang S, Keck M, Przibilla M, Lamb DJ, Schiele F, Wittenbrink M, Fuchs K, Jung B, Erb KJ, Peter D (2017). Neutralization of both IL-1α/IL-1β plays a major role in suppressing combined cigarette smoke/virus-induced pulmonary inflammation in mice. Pulm Pharmacol Ther.

[13] Kim KS, Jung H, Shin IK, Choi BR, Kim DH (2015). Induction of interleukin-1 beta (IL-1β) is a critical component of lung inflammation during influenza A (H1N1) virus infection. J Med Virol.

[14] Hughes JE, Srinivasan S, Lynch KR, Proia RL, Ferdek P, Hedrick CC (2008). Sphingosine-1-phosphate induces an antiinflammatory phenotype in macrophages. Circ Res.

[15] Walsh KB, Teijaro JR, Wilker PR, Jatzek A, Fremgen DM, Das SC, Watanabe T, Hatta M, Shinya K, Suresh M, Kawaoka Y, Rosen H, Oldstone MB (2011). Suppression of cytokine storm with a sphingosine analog provides protection against pathogenic influenza virus. Proc Natl Acad Sci U S A.

[16] Weichand B, Popp R, Dziumbla S, Mora J, Strack E, Elwakeel E, Frank AC, Scholich K, Pierre S, Syed SN, Olesch C, Ringleb J, Ören B, Döring C, Savai R, Jung M, von Knethen A, Levkau B, Fleming I, Weigert A, Brüne B (2017). S1PR1 on tumor-associated macrophages promotes lymphangiogenesis and metastasis via NLRP3/IL-1β. J Exp Med.

[17] Tantikanlayaporn D, Tourkova IL, Larrouture Q, Luo J, Piyachaturawat P, Witt MR, Blair HC, Robinson LJ (2018). Sphingosine-1-phosphate modulates the effect of estrogen in human osteoblasts. JBMR Plus.

[18] Tian T, Tian W, Yang F, Zhao R, Huang Q, Zhao Y (2016). Sphingosine kinase 1 inhibition improves lipopolysaccharide/D-galactosamine-induced acute liver failure by inhibiting mitogen-activated protein kinases pathway. United Eur Gastroenterol J.

[19] Li Y, Xie P, Sun M, Xiang B, Kang Y, Gao P, Zhu W, Ning Z, Ren T (2016). S1PR1 expression correlates with inflammatory responses to Newcastle disease virus infection. Infect Genet Evol.

[20] Tong S, Tian J, Wang H, Huang Z, Yu M, Sun L, Liu R, Liao M, Ning Z (2013). H9N2 avian influenza infection altered expression pattern of sphiogosine-1-phosphate receptor 1 in BALB/c mice. Virol J.

[21] Kai Y, Hu Z, Xu H, Hu S, Zhu J, Hu J, Wang X, Liu X, Liu X (2015). The M, F and HN genes of genotype VIId Newcastle disease virus are associated with the severe pathological changes in the spleen of chickens. Virol J.

[22] Cannon G, Callahan MA, Gronemus JQ, Lowy RJ (2014). Early activation of MAP kinases by influenza A virus X-31 in murine macrophage cell lines. PLoS ONE.

[23] Cheng SC, Huang WC, Pang SJH, Wu YH, Cheng CY (2019). Quercetin inhibits the production of IL-1β-induced inflammatory cytokines and chemokines in ARPE-19 cells via the MAPK and NF-κB signaling pathways. Int J Mol Sci.

[24] Pan H, Zhang Y, Luo Z, Li P, Liu L, Wang C, Wang H, Li H, Ma Y (2014). Autophagy mediates avian influenza H5N1 pseudotyped particle-induced lung inflammation through NF-κB and p38 MAPK signaling pathways. Am J Physiol Lung Cell Mol Physiol.

[25] Walsh KB, Teijaro JR, Brock LG, Fremgen DM, Collins PL, Rosen H, Oldstone MBA (2014). Animal model of respiratory syncytial virus: CD8+ T cells cause a cytokine storm that is chemically tractable by sphingosine-1-phosphate 1 receptor agonist therapy. J Virol.

[26] Teijaro JR, Walsh KB, Cahalan S, Fremgen DM, Roberts E, Scott F, Martinborough E, Peach R, Oldstone MB, Rosen H (2011). Endothelial cells are central orchestrators of cytokine amplification during influenza virus infection. Cell.

[27] Teijaro JR, Walsh KB, Long JP, Tordoff KP, Stark GV, Eisfeld AJ, Kawaoka Y, Rosen H, Oldstone MBA (2014). Protection of ferrets from pulmonary injury due to H1N1 2009 influenza virus infection: Immunopathology tractable by sphingosine-1-phosphate 1 receptor agonist therapy. Virology.

[28] Teijaro JR, Walsh KB, Rice S, Rosen H, Oldstone MBA (2014). Mapping the innate signaling cascade essential for cytokine storm during influenza virus infection. Proc Natl Acad Sci U S A.

[29] Xu XQ, Huang CM, Zhang YF, Chen L, Cheng H, Wang JM (2016). S1PR1 mediates anti-apoptotic/pro-proliferative processes in human acute myeloid leukemia cells. Mol Med Rep.

[30] Doyle TM, Chen Z, Durante M, Salvemini D (2019). Activation of sphingosine-1-phosphate receptor 1 in the spinal cord produces mechanohypersensitivity through the activation of inflammasome and IL-1β pathway. J Pain.

[31] Lauro F, Giancotti LA, Kolar G, Harada CM, Harmon TA, Garrett TJ, Salvemini D (2021). Role of adenosine kinase in sphingosine-1-phosphate receptor 1-induced mechano-hypersensitivities. Cell Mol Neurobiol.

[32] Takahashi M (2022). NLRP3 inflammasome as a key driver of vascular disease. Cardiovasc Res.

